# Biodiesel production potential of *Eichhornia crassipes* (Mart.) Solms: comparison of collection sites and different alcohol transesterifications

**DOI:** 10.1038/s41598-024-51913-y

**Published:** 2024-01-18

**Authors:** Aricely Aparecida Silva Leite, Luciana Vincenzi Weber, João Paulo Aquino Correa, Thiago Luis Aguayo de Castro, Carmem Cícera Maria da Silva, Rosangela Maria Ferreira da Costa e Silva, Claudia Andrea Lima Cardoso, Leila Cristina Konradt-Moraes

**Affiliations:** 1 State University of Mato Grosso do Sul (UEMS), Dourados, Mato Grosso do Sul 79804-970 Brazil; 2 Chemistry Department, Federal University of Espírito Santo (UFES), Campus Goiabeiras, Vitória, Espírito Santo 29075-910 Brazil

**Keywords:** Environmental sciences, Energy

## Abstract

Renewable resources have stood out as raw materials in producing biofuels. This study aimed to evaluate the parameters of alcohol transesterification (ethanol and methanol) and localization of collection of aquatic macrophyte *Eichhornia crassipes* (Mart.) Solms in the production of biodiesel by in situ transesterification. *E. crassipes* was collected in Dourados and Corumbá (Brazil) municipalities. The fatty acid ester composition of the biodiesel was characterized and quantified by gas chromatography. The biodiesel properties were estimated using the BiodieselAnalyzer© program prediction. The ethyl transesterification resulted in higher yields, but the localization of collection was the most relevant parameter in biodiesel production according to the Permutation Multivariate Analysis of Variance. The simulation and comparison of the physical–chemical properties of *E. crassipes* biodiesel and BD 100 (commercial biodiesel) were promising for commercial application.

## Introduction

The world's diesel consumption reached 3.9 million tons in 2019^1^. Diesel is a non-renewable energy matrix from petroleum with a high impact on climate change due to emissions of pollutants such as carbon dioxide (CO_2_), carbon monoxide (CO), sulfur dioxide (SO_2_), and nitrogen oxide (NO_X_)^2^.

Hundreds of countries signed the Paris Agreement and agreed to aim at “holding the increase in the global average temperature to well below 2 °C above pre-industrial levels". The Paris Agreement established that each country is responsible for planning, communicating, and implementing Nationally Determined Contributions to reduce emissions^3^. One of Brazil's strategies for reducing emissions of pollutants is the increasing replacement of diesel by biodiesel. The addition of biodiesel in commercial Brazilian diesel reached 12% in 2023, and the demand for biodiesel could achieve 9 billion liters in 2024 to accomplish the objectives of substitution of 15% of diesel (https://www.in.gov.br/en/web/dou/-/despacho-do-presidente-da-republica-473383252)^2,4^.

Biodiesel is non-toxic, biodegradable, and sulfur-free and has a low carbon footprint. Life cycle assessment studies show that biodiesel can reduce carbon monoxide (CO) and carbon dioxide (CO_2_) emissions by 8–41%^5^. However, the worldwide consumption of biodiesel has been limited by the cost of feedstock and feedstock sources that include oilseeds such as soybean, corn, peanut, and sunflower, which have nobler applications such as human and animal food^4–6^. Given the growing demand for food and biodiesel, obtaining new sources of biodiesel becomes increasingly necessary for the growth of the biodiesel market^4,7^.

*Eichhornia crassipes* (Mart.) Solms are native macrophytes of Sul of America with a high capacity of propagation and adaptation tolerance to diverse environments, and a high growth rate in eutrophicated media turns this aquatic plant into a potential biomass source to produce biofuels^8–10^. Another exciting application of these aquatic macrophytes is in the phytoremediation process due to their ability to accumulate metals, act in carbon sequestration in limnic environments, and provide substrate for periphytic/bacterial communities^10–12^. The association of these capacities results in two gains: producing sustainable biodiesel and eliminating pollution in rivers, lakes, and other aquatic rooms^13–15^.

To increase biodiesel production's economic, social, and environmental viability using this macrophyte, variables such as the transesterification process and choice of alcohol type must be evaluated. Biodiesel can be produced through oil or fat previously extracted (conventional transesterification) or direct (in situ) transesterification, which performs the chemical reaction directly on the biomass^9,16^. Direct transesterification reduces production costs, process time, and losses due to eliminating oil extraction steps before transesterification^17^. The main alcohols used in the reaction of transesterification of lipids into fatty acid esters are methanol and ethanol^18^. Transesterification using methanol is the world's most used in biodiesel production due to the cost, but methanol is of fossil origin. However, the ethanol of sugarcane is widely produced in Brazil and, therefore, a local and ecological option for biodiesel production^4,19^.

Some *E. crassipes* biodiesel parameters, such as the impact of seasons of the year in biodiesel compositions, performance, the influence of injection timing and load on the performance, combustion, and emission characteristics for substitution of 10–100% of petroleum diesel using conventional transesterification and methanol have been studied by other researchers^8,13,20,21^. However, the evaluation of the collection environment and alcohol used in chemical composition and physical–chemical proprieties is not explored in the literature.

In this work, we evaluate the influence of the collection site and the use of methanol and ethanol as alcohol types in *E. crassipes* biodiesel production by in situ transesterification. The composition of fatty acid esters and biodiesel unsaturation parameters of the *E. crassipes* biodiesel has been compared to the commercial biodiesel (BD 100) and the diverse literature. Multivariate data analysis was realized, and the BiodieselAnalyzer© program was used to estimate the physicochemical properties of biodiesel samples.

## Results and discussion

**Figure 1:**
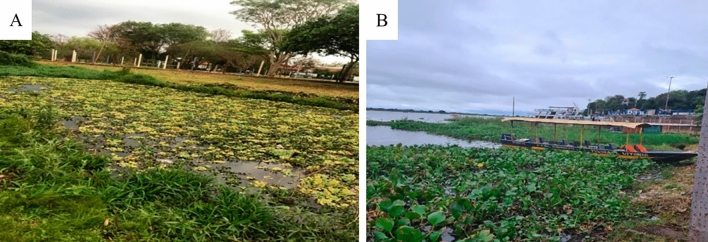
Macrophyte collection point: (**A**) Arnulpho Fioravante Park, city of Dourados (Brazil); and (**B**) macrophyte collection point at Porto Geral de Corumbá, city of Corumbá (Brazil).

*E. crassipes* samples were collected in distinct biomes: Corumbá harbor is in the Paraguay River, which presents a large extension and high flow velocity, unlike the lagoon of Dourados, which offers a lentic ecosystem and minor extension. Visually, the leaves of *E. crassipes* of Dourados samples (Fig. 1A) were smaller than the Corumbá samples (Fig. 1B).

**Figure 2:**
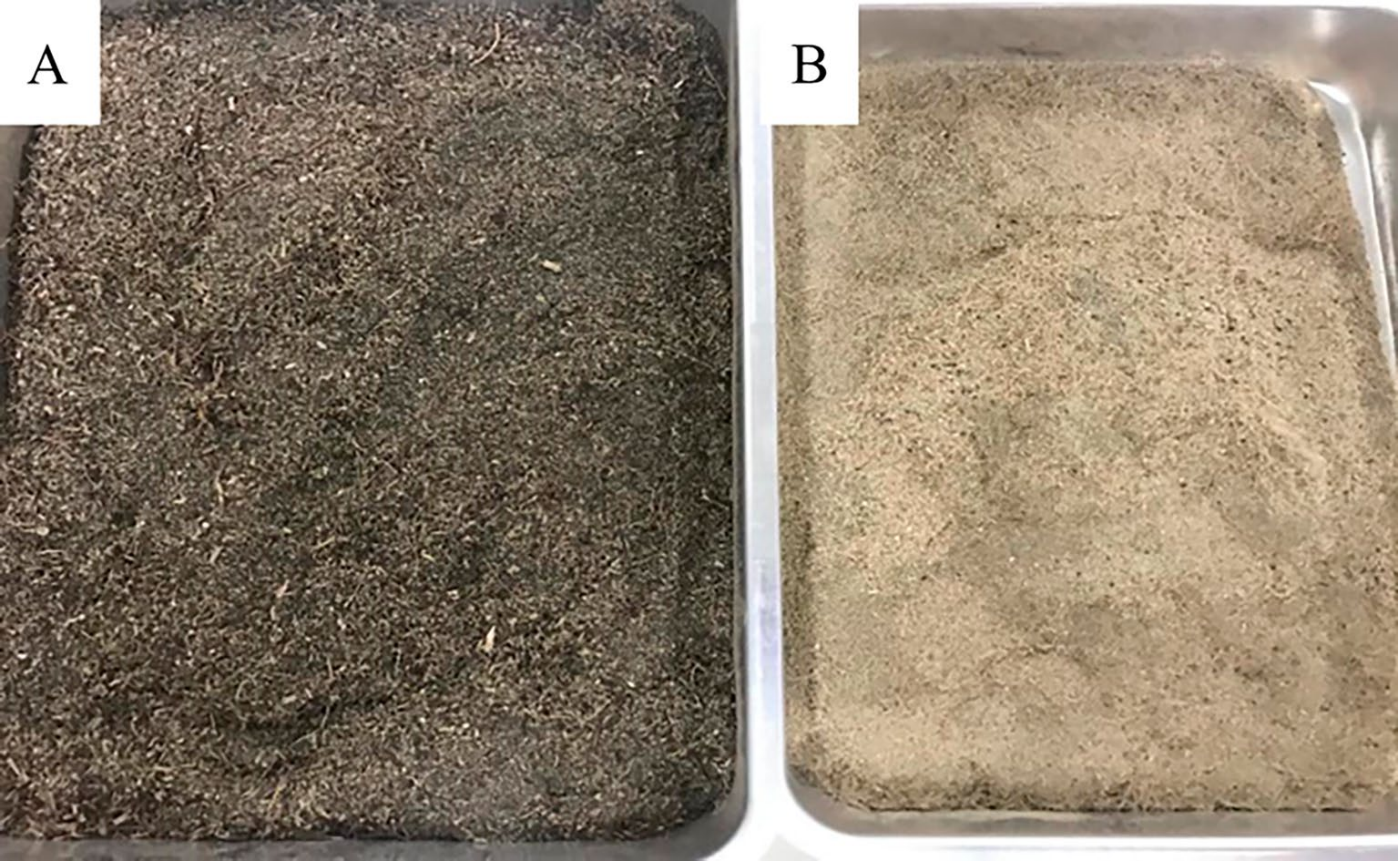
Aspect of processed biomass collected in the cities of (**A**) Dourados (Brazil) and (**B**) Corumbá (Brazil) samples.

The dried biomasses presented different colors (Fig. 2). The predominance of dark colors from the Dourados sample (2A) can be attributed to the excess ammonia in the aquatic environment. Shengqi et al*.*^22^ reported that excess ammonia could trigger oxidative stress in macrophyte tissues, as indicated by increased antioxidant enzymes that may be partially responsible for decreased growth. Therefore, aquatic macrophytes should exhibit higher rates of primary production (synthesis of organic matter from inorganic compounds) in environments with low ammonia concentrations in the water.

This observation explained the initial incoherence in the size of the leaves of *E. crassipes*. Macrophyte biomass is usually abundant in water with low velocity, sediment stability, high temperatures, and nutrient availability^23,24^. Therefore, the Dourados sample can have higher favorable conditions. However, the excess of ammonia caused an imbalance in environmental conditions.

**Figure 3:**
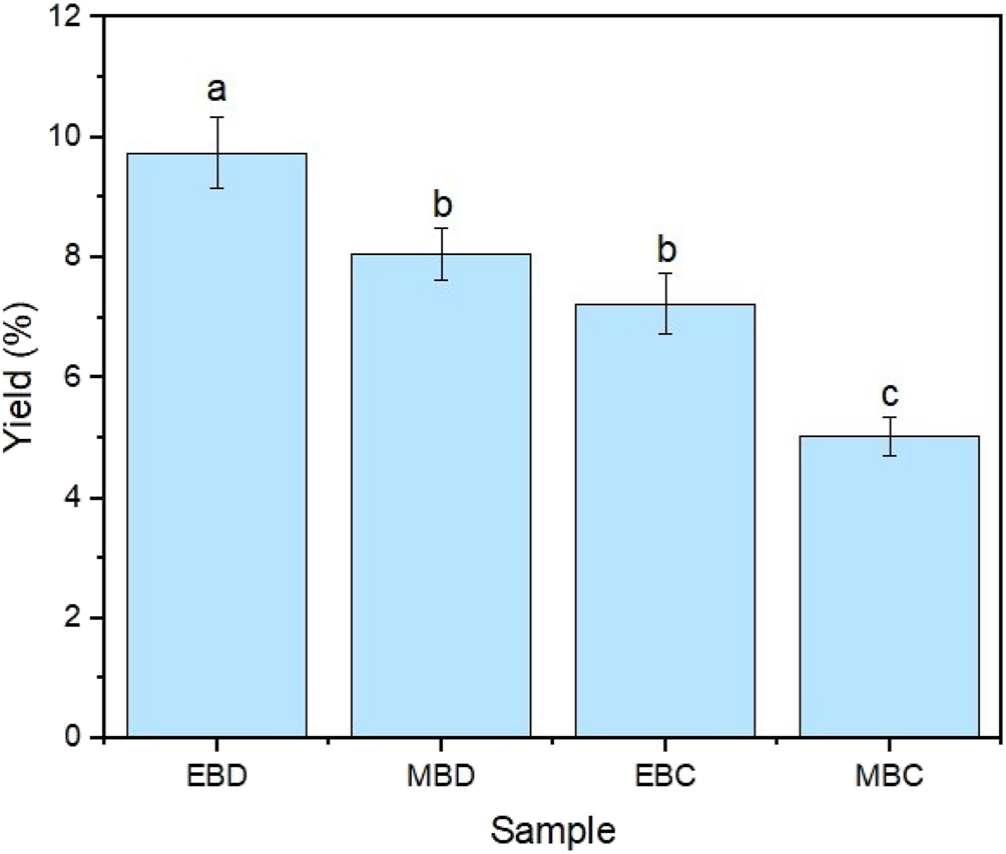
Yield of biodiesel production. *EBD* Dourados ethyl biodiesel, *MBD* Dourados methyl biodiesel, *EBC* Corumbá ethyl biodiesel, *MBC* Corumbá methyl biodiesel.

The yield of the biodiesel production by ethanol transesterification (EB, 9.74 ± 0.59) was higher than methanol transesterification (MB, 8.06 ± 0.43) for Dourados and Corumbá samples (Fig. 3). The parameter local shows that although the leaves of plants Dourados are minor (Fig. 1A, B) the yield of biodiesel of the EBD (Ethyl biodiesel with *E. crassipes* from Dourados) and MBD (Methyl biodiesel with *E. crassipes* from Dourados) transesterification was higher considerable than EBC (Ethyl biodiesel with *E. crassipes* from Corumbá) and MBC (Methyl biodiesel with *E. crassipes* from Corumbá) transesterification. The yields found in this work are higher than those described in the literature by Shanab et al., which obtained biodiesel yields ranging from 3.3 to 6.4% for the fourth different seasons of the year, using methanol and the traditional transesterification^8^. The environmental factor directly affects the lipid content and the yield of direct transesterification, as demonstrated by Oliveira Junior et al.^10^, who grew the macrophyte *Salvinia auriculata* Aubl. with vinasse and biosolids, a difference was found in the extracted oil yield and direct transesterification.

The minor concentration of lipids in Corumbá samples (7.23 ± 0.51, 5.02 ± 0.31, for EBC and MBC, respectively) can be explained by the higher and constantly renewed water volume compared to Dourados. Considering its characteristics, the Paraguay River has an easier self-depuration and dilution of the organic load, probably generating a lower burden of compounds absorbed by the local plants. Dourados' nutrients tend to accumulate more due to a lacustrine environment. Therefore, the plant has more nutrients available locally.

The ANOVA and Tukey tests' statistical evaluation of biodiesel yield (Fig. 3) shows that the alcohol and collection site used resulted in a significant difference in biodiesel yield by the ANOVA and Tukey tests. Different letters indicate a significant difference (p < 0.05), while equal letters indicate that it is not possible to say that there is a substantial difference between the samples (p > 0.05). The results suggest that both factors directly influence the yield of biodiesel produced, with ethanol being the best alcohol and the city of Dourados being the best collection location.

The variation of fatty acid ester in the composition of biodiesel will determine the biodiesel's physical and chemical characteristics, such as oxidative stability, clogging point, and cetane number, among others^25^. Using methanol or ethanol in biodiesel production (Table 1) does not significantly differ in fatty acid ester content compositions. However, the collection site shows a considerable variation in fatty acid ester composition. The EBC and MBC samples resulted in three times higher linolenic ester concentrations and minor concentrations of all other esters than EDB and MBD. The evaluation composition of esters obtained experimentally of *E. crassipes* biodiesel (Table 1) by PERMANOVA indicated that the location of collection (pseudo-*F* ratio = 2844.53; p < 0.001) was more relevant than the alcohol type used in transesterification (pseudo-F ratio = 4.46; p < 0.050). These facts corroborate the literature and confirm the relevance of environmental factors in the biodiesel composition of *E. crassipes*^8^.

**Table 1:** Composition of fatty acid esters in biodiesels.

Molecule		Fatty esters composition				
EAG	Corresponding fatty acid	BD 100 (%)	EBD (%)	MBD (%)	EBC (%)	MBC (%)
C16:0	Palmitic acid	15.76 ± 0.13^c^	25.17 ± 0.13^a^	25.34 ± 0.11^a^	23.31 ± 0.39^b^	23.81 ± 0.16^b^
C16:1	Palmitoleic acid	0.23 ± 0.01^c^	5.32 ± 0.06^a^	5.36 ± 0.04^a^	0.31 ± 0.02^bc^	0.36 ± 0.02^b^
C18:0	Stearic acid	6.01 ± 0.16^b^	7.22 ± 0.07^a^	7.31 ± 0.09^a^	5.29 ± 0.05^c^	5.48 ± 0.12^c^
C18:1	Oleic acid	19.13 ± 0.24^c^	27.13 ± 0.28^a^	27.41 ± 0.17^a^	24.11 ± 0.11^b^	24.00 ± 0.10^b^
C18:2	Linoleic acid	46.34 ± 0.42^a^	29.96 ± 0.18^b^	28.30 ± 0.18^c^	28.45 ± 0.49^c^	27.43 ± 0.15^d^
C18:3	Linolenic acid	8.99 ± 0.14^b^	3.98 ± 0.06^c^	4.01 ± 0.12^c^	14.82 ± 0.19^a^	14.99 ± 0.09^a^

*EAG* fatty acid esters, *BD 100* fuel containing 100% commercial biodiesel in its composition, *EBD* Dourados ethyl biodiesel, *MBD* Dourados methyl biodiesel, *EBC* Corumbá ethyl biodiesel, *MBC* Corumbá methyl biodiesel.

Different letters indicate significant differences by the Tukey test at a 5% significance level in line.

**Figure 4:**
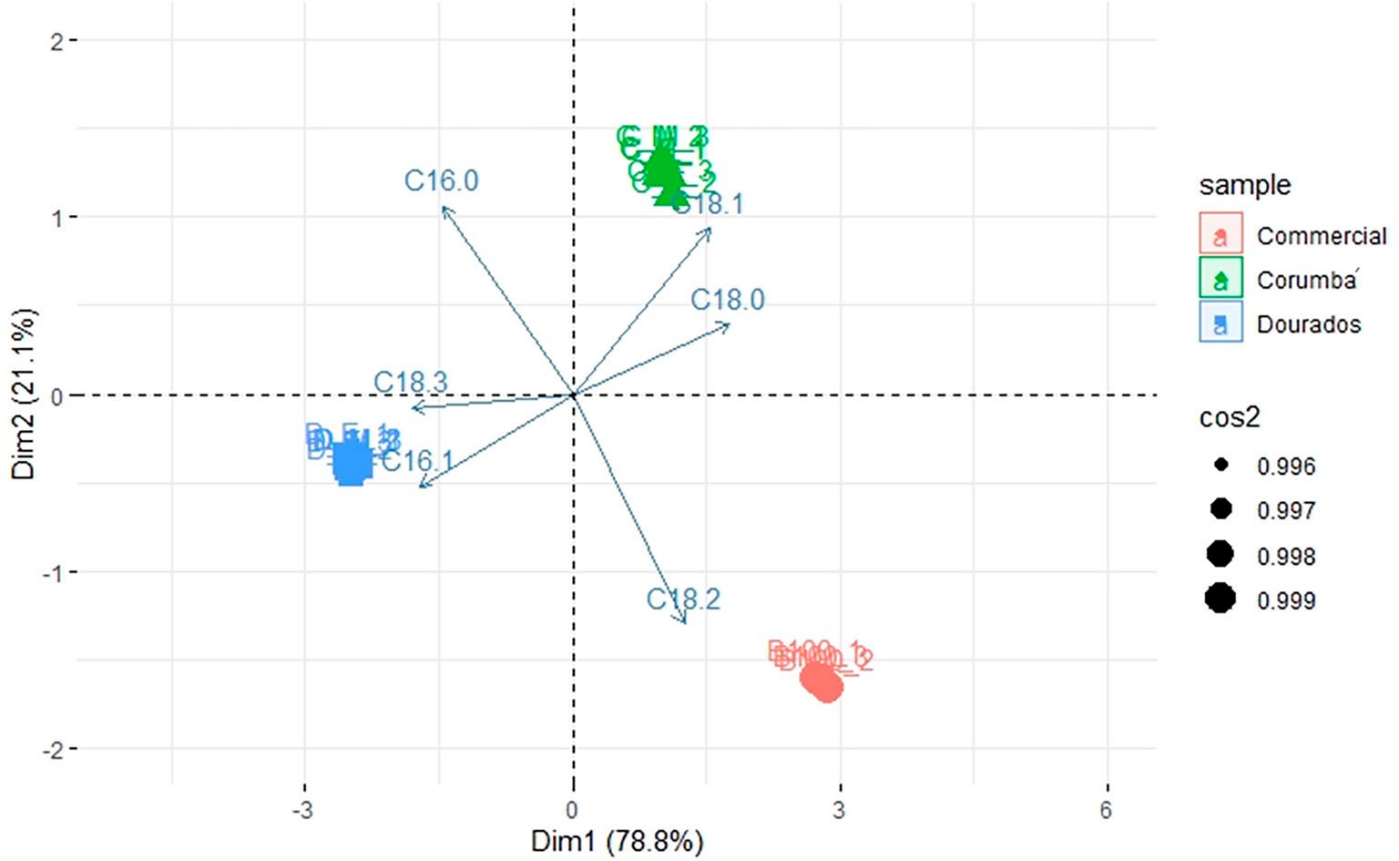
Principal component analysis (PCA) of the chemical composition of biodiesels.

The comparison of ester contents determined experimentally with BD 100 (commercial biodiesel) showed distinct concentrations of esters for the three biodiesel samples. Principal component analysis (PCA) of the chemical composition of biodiesels of *E. crassipes* was performed in R platform, explaining 99.9% of data (Fig. 4). The graphical indicated a separation of samples by collection site, mainly related by C16:1, C18:0, C18:1 and C18:3 compounds (Fig. 4). The C16:0 and C18:2 fatty acid esters composition were essentials for commercial biodiesel separation from the Corumbá and Dourados biodiesel samples. The C16:0 is lower in BD 100 (commercial sample) than in other samples, while C18:2 is higher. Therefore, these compounds are the main differentiators between commercial Brazilian biodiesel and *E. crassipes* biodiesel.

The comparison of the fatty acid ester composition of the *E. crassipes* of this work with *E. crassipes* biodiesel prepared by other researchers and other biodiesel types described in the literature shows significant variations in the compositions, too. Shanab et al. evaluated the variation of ester concentration of biodiesel of *E. crassipes* as a function of seasons year by traditional transesterification. The studies indicated the composition variations for each season, and the esters' compositions were distinct from the experimental data in this paper. The concentration of stearic acid methyl ester was 69%, and myristic acid methyl ester was 12% in the summer. In the autumn and winter, the concentration of myristic acid methyl ester was 100% for oil extraction by methanol/chloroform^8^.

**Figure 5:**
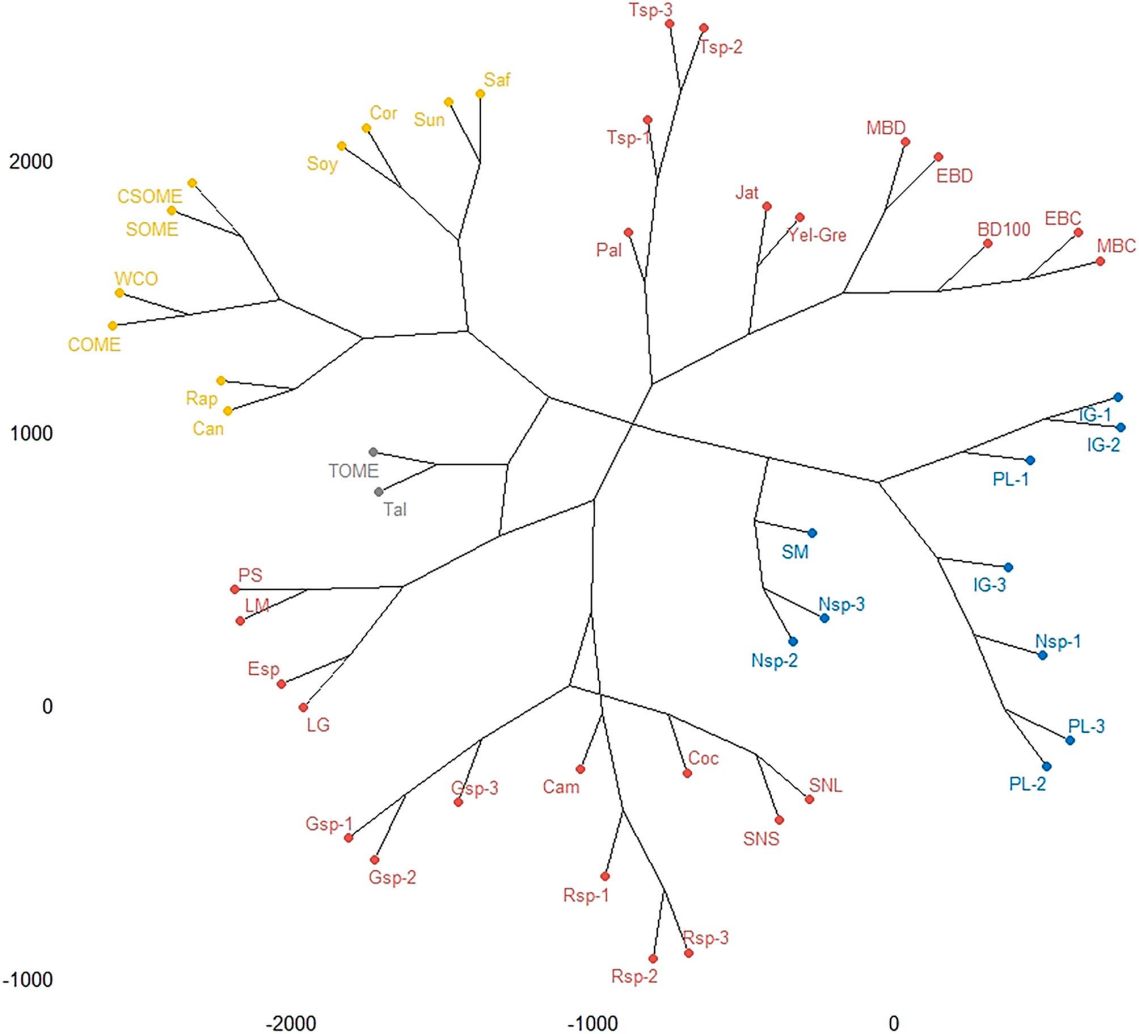
Cluster dendrogram of symmetry of the samples by a chemical composition by Euclidean distance. *BD 100* commercial biodiesel, *EBD* ethyl biodiesel with *E. crassipes* from Dourados, *MBD* methyl biodiesel with *E. crassipes* from Dourados, *EBC* ethyl biodiesel with *E. crassipes* from Corumbá, *MBC* methyl biodiesel with *E. crassipes* from Corumbá, SM *Salvinia molesta*^9^; *SOME* soybean oil, *WCO* waste cooking oil, *CSOME* cotton oil, *COME* canola oil^26^, IG *Isochrysis galbana*; PL *Pavlova lutheri*; Gsp *Gymnodinium* sp; Nsp *Nannochloropsis* sp; Tsp *Tetraselmis* sp; Rsp *Rhodomonas* sp^27^; LG *L. gibba*; PS *Pistia stratiotes*; LM *L. minor*; Esp *Eichhornia* sp^28^; SNL *Salvina natans* leaves; SNS *Salvinia natans* sorus^29^; *Cam* camelina, *Can* canola, *Coc* coconut, *Cor* corn, *Jat* jatropha, *Pal* palm, *Rap* rapeseed, *Saf* safflower, *Soy* soy, *Sun* sunflower, *Tal* tallow, *Yel-Gre* yellow grease^38^.

The Euclidean distance was used to evaluate the similarity of the ester’s composition of experimental samples and several commercial and non-commercial biodiesels described in the literature^9,26–29,38^. The cophenetic correlation analysis of the fatty acid content was 0.7722636. Comparing the dendrogram of the biodiesels (Fig. 5), the percentage amounts of esters and the type of the esters determined experimentally showed many variations when compared to *E. crassipes* biodiesel and between the diverse commercial biodiesels described in the literature. The composition of *E. crassipes* biodiesel was more similar to samples of the Jat (Jatropha) and Yel-Gre (Yelow grease) biodiesel`s. The Jat is a plant, and the denomination Yellow grease is used for cooking oil. Variations in the composition of the esters can be mainly attributed to the source of the oil, the place of collection, the period of the year in which the raw material was collected, the processes and solvents used in extracting the oils, and the type of transesterification process^9,16,26–29,38^.

Despite variations between the components and % composition of *E. crassipes* biodiesel in relation to the literature for biodiesel from the same source (*E. crassipes*) and other sources, the components of *E. crassipes* biodiesel found in the experiment corroborate the study of Jahirul et al. that evaluated 125 paper and conclude that the main fatty acid ester composition in % of the occurrence are: oleic (C18:1); stearic (18:0), linoleic (C18:2), palmitic (C16:0) and linolenic (C18:3) for commercial and non-commercial biodiesel in the literature. The occurrence of the fatty acid esters oleic, linoleic, palmitic, linolenic, and stearic acid in the different biodiesel samples was 40, 32, 16, 7.5, and 6.5%, respectively^26^.

The determinants properties of biodiesel, such as viscosity, the heat of combustion, oxidative stability, ignition quality, and lubricity, are influenced by the composition percentual of the fatty esters, the unsaturation of chains, and the ester moiety derived from the alcohol comprising biodiesel^25^. However, the dendrogram shows the diverse esters compositions of the commercial biodiesel samples described in the literature. Therefore, the ester compositions of biodiesels are determinants in the physicochemical parameter, but several esters' compositions produce viable physicochemical properties for commercial biodiesel.

Analyzing the unsaturated parameters of biodiesels of *E. crassipes* shown in Table 2, the MUFA/PUFA (mono-unsaturated/poly-unsaturated fatty acids) ratio of the MBD and EBD biodiesels is approximately one, i.e., the composition of the monounsaturated and polyunsaturated compounds are the like. MBC and EBC biodiesel and BD 100 resulted in values next to 0.6 and 0.35, respectively, indicating the predominance of PUFA. According to Stansell et al., using monounsaturated compounds results in better proprieties, such as the kinematic viscosity of biodiesel^30^.

**Table 2:** Biodiesel unsaturation parameters.

Sample	BD 100	EBD	MBD	EBC	MBC
SFA (%)	25.29 ± 0.02^d^	34.61 ± 0.05^a^	34.92 ± 0.20^a^	32.45 ± 0.42^c^	33.32 ± 0.19^b^
MUFA (%)	19.36 ± 0.23^c^	32.45 ± 0.34^a^	32.77 ± 0.21^a^	24.40 ± 0.10^b^	24.33 ± 0.11^b^
PUFA (%)	55.33 ± 0.56^a^	33.94 ± 0.24^c^	32.31 ± 0.30^d^	43.48 ± 0.51^b^	42.40 ± 0.24^b^
MUFA/PUFA	0.350 ± 0.001^d^	0.956 ± 0.003^b^	1.014 ± 0.003^a^	0.562 ± 0.005^c^	0.572 ± 0.005^c^
ANC	17.65 ± 0.15^a^	17.571 ± 0.098^a^	17.39 ± 0.13^a^	17.593 ± 0.038^a^	17.527 ± 0.004^a^
ANDB	1.390 ± 0.015^a^	1.043 ± 0.009^c^	1.014 ± 0.009^d^	1.264 ± 0.012^b^	1.241 ± 0.005^b^

*BD 100* fuel containing 100% biodiesel in its composition, *EBD* Dourados ethyl biodiesel, *MBD* Dourados methyl biodiesel, *EBC* Corumbá ethyl biodiesel, *MBC* Corumbá methyl biodiesel, *SFA* saturated fatty acid (%), *MUFA* monounsaturated compounds, *PUFA* polyunsaturated compounds, *ANC* the average number of carbons, *ANDB* average number of double bonds.

Different letters indicate significant differences by the Tukey test at a 5% significance level.

The average number of carbons (ANC), denominated by average chain length for other literature, was statically the same and corroborated with the biodiesel literature^26^. The average number of double bonds (ANDB) of BD e BC samples was 25 and 10% smaller than for the BD 100 sample. ANDB to have a strong influence on all biodiesel characteristics^26^. Jahirul et al. established the superior limit of 2 for accomplishing biodiesel standards in terms of cetane number (CN), iodine value (IV), oxidation control, and Cold filter plugging pint temp (CFPP)^26^. The increase in carbon chain length results in an increase in kinematic viscosity (υ). Kinematic viscosity (υ) and density (ρ) determine fuel performance properties for diesel engines as fuel spraying, fuel–air mixture, and combustion. The incomplete combustion produces gas emissions such as carbon monoxide (CO) and unburnt hydrocarbon (UHC)^6,9,26^.

The correct balance between monounsaturated fatty acids (MUFA) concentrations and the total saturated fatty acid (SFA) composition results in better proprieties and higher performance biodiesel^30^. The MBD and EBD biodiesel showed a higher percentage of saturated (SFA) and monounsaturated (MUFA) and minor concentrations of polyunsaturated (PUFA) ester as compared to the BD 100 (Table 2). In low temperatures, biodiesel has been causing clogging of filters or restricting the flow during ignition due to crystallization by excess saturated fatty acid esters (SFA). The high percent of SFA increases the melting point, giving the high biofuel values of Cloud Point (CP), Cold Filter Plugging Point (CFPP), and Pour Point (PP)^25^. The presence of one or more unsaturation in the monoalkyl esters of fatty acids with saturated hydrocarbon chains of C16 (palmitic) and C18 (stearic) reduces the tendency to solidify at low temperatures^31^, reduces the viscosity of the biodiesel and improves the filter-to-filter plugging point properties. However, an excess of unsaturated fatty acids, especially PUFA, will cause low oxidative stability in the biofuel^25^.

The physicochemical proprieties are determined by fatty acid ester composition, alcohol type, and many other parameters. For the easy evaluation of the bioprospecting of new sources of biodiesel production, some software has been available that estimates with good concordance the physicochemical proprieties of biodiesel based on the chemical composition^23^.

BiodieselAnalyzer© was already used by the simulation of physicochemical proprieties of biodiesel from the fungus *Mucor circinelloides* produced with vinasse^32^, from the plant *Prunus avium* (L.) L., from the microalgae *Chlorella*^33^, and the yeast *Rhodosporidium toruloides*^34^. The simulation of the physicochemical proprieties of biodiesel samples of this work using the BiodieselAnalyzer© is shown in Table 3. Evaluating the individual proprieties as cetane number (CN), density (ρ), calorific power value (HHV), oxidative stability (OS), and unsaturation degree (UD) have observed parameters that are favorable and others that can be improved by control of the production process ^13^. CN will determine the time between the fuel injection into the cylinder and the ignition start. High values of CN result in minor ignition delay, good cold start proprieties, and reduced white smoke formation^26^. *E. crassipes* biodiesel (MBC, MBD, EBC, EBD) should have higher ignition speeds than commercial sample BD 100.

**Table 3:** Estimated biodiesel properties.

	ANP ^23^	ASTM ^24^	EN ^25^	BD 100	EBD	MBD	EBC	MBC	^14^
UD	–	–	–	130.02 ± 1.4^a^	100.33 ± 0.82^c^	97.39 ± 0.81^d^	111.36 ± 1.1^b^	109.07 ± 0.41^b^	–
VS	194	–	–	203.08 ± 1.5^b^	207.19 ± 1.0^a^	205.23 ± 1.4^ab^	204.81 ± 0.31^ab^	204.35 ± 0.04^b^	41
VI	< 120	< 47	< 120	125.98 ± 1.4^a^	94.87 ± 0.80^c^	92.24 ± 0.85^d^	114.65 ± 1.1^b^	112.53 ± 0.45^b^	–
CN	< 60	–	< 51	44.83 ± 0.51^c^	51.30 ± 0.31^a^	52.14 ± 0.37^a^	47.14 ± 0.28^b^	47.69 ± 0.10^b^	48
LCSF	–	–	–	6.92 ± 0.12^c^	7.82 ± 0.04^b^	7.91 ± 0.06^b^	8.28 ± 0.10^a^	8.37 ± 0.04^a^	–
CFPP	9	Report	–	5.27 ± 0.38^c^	8.08 ± 0.13^b^	8.39 ± 0.19^b^	9.55 ± 0.33^a^	9.83 ± 0.12^a^	–
PN	–	−15 to 10	–	3.30 ± 0.07^c^	8.25 ± 0.07^a^	8.34 ± 0.06^a^	7.21 ± 0.21^b^	7.52 ± 0.09^b^	–
PF	–	> 3	–	−3.24 ± 0.07^c^	2.13 ± 0.07^a^	2.23 ± 0.06^a^	1.00 ± 0.23^a^	1.34 ± 0.09^b^	17
OS	> 12	–	> 6	4.72 ± 0.02^d^	6.07 ± 0.02^b^	6.24 ± 0.03^a^	5.30 ± 0.03^a^	5.37 ± 0.02^c^	–
HHV	–	1.9–6	–	39.38 ± 0.32^a^	39.80 ± 0.21^a^	39.40 ± 0.28^a^	39.50 ± 0.07^a^	39.38 ± 0.01^a^	42.7
υ	3–6	8.5–9	3.5–5	3.60 ± 0.04^b^	3.79 ± 0.03^a^	3.75 ± 0.03^a^	3.65 ± 0.01^b^	3.64 ± 0.01^b^	26.4
ρ	–	–	0.87	0.88 ± 0.01^a^	0.89 ± 0.01^a^	0.88 ± 0.01^a^	0.88 ± 0.01^a^	0.88 ± 0.01^a^	0.95

*BD 100* biodiesel common, *EBD* ethyl biodiesel Dourados, *MBD* methyl biodiesel Dourados, *EBC* ethyl biodiesel Corumbá, *MBC* methyl biodiesel Corumbá, *UD* unsaturation degree, *VS* saponification value (mg KOH g^−1^), *VI* iodine value (mg g^−1^), *CN* cetane number, *LCSF* saturated long-chain factor, *CFPP* cold filter plugging point (°C), *PN* cloud point (°C), *PF* pour point (°C), *OS* oxidation stabilization (h), *HHV* calorific value (MJ kg^−1^), *υ* kinematic viscosity (mm^2^ s^−1^), *ρ* density (g cm^−3^), *ANP* Agência Nacional do Petróleo, Gás Natural e Biocombustíveis, *ASTM* American Society for Testing and Materials, EN European Norm.

Different letters indicate significant differences by the Tukey test at a 5% significance level.

The density is related to the CN and the calorific value^35^. The calorific value determines thermal efficiency. That is, the lower the calorific value of the fuel, the higher the consumption to release the same amount of energy^36^. Experimental biodiesel samples and BD 100 showed the same performance. Venu et al. obtained an HHV value of 35.80 MJ kg^−1^ for *E. crassipes* under different conditions^20^. The calorific power value (HHV) of biodiesels of E. crassipes was 10% less than that of petroleum diesel (45 MJ kg^−1^); therefore, the HHV value is considered adequate for commercial biodiesel^26^. OS of *E. crassipes* biodiesel, mainly EBD and MBD, was higher than BD 100 (Table 3), indicating better performance for EBD and MBD biodiesel. Wahhab and Al-Kayiem studied the effect of adding *E. crassipes* biodiesel in diesel and observed an increase in FP and NC and a decrease in PP^13^.

The unsaturation degree (UD) of EBD and MBD was approximately 30% less than that of BD 100. The smaller UD value is a good indicator for commercial biodiesel. The simulated results obtained by BiodieselAnalyzer© (Table 3) corroborate with the MUFA/PUFA relation and other biodiesel unsaturation parameters (Table 2) determined by experimental data.

## Conclusions

Almost all parameters of the experimental biodiesel samples (Table 2) and simulated physicochemical proprieties by BiodieselAnalyzer© (Table 3) are within the limits allowed by Brazilian legislation (by ANP) or are many times better than the values found for the commercial sample (BD 100). Comparing the experimental data and literature, the collection site, alcohol type, and the transesterification process greatly impacted the biodiesel composition. Ethanol is a better alcohol type and is locally more favorable due to its high availability, reduction of environmental effects, and higher safety for use as reagents in biodiesel production.

The *E. crassipes* biodiesel of Dourados (EBD) shows superiority in some evaluated parameters, and the collecting environment has higher quantities of organic material (eutrophicated environment), resulting in biodiesel rich in monounsaturated fatty acid ester, which is favorable for less viscosity and higher stability of biodiesel. Therefore, the use of *E. crassipes* as a source of oil by biodiesel production associated with the bioremediation of organic pollution of aquatic is a potential alternative for the solution of two big problems in developing countries.

## Materials and methods

### Collecting *E. crassipes*

Two cities in Mato Grosso do Sul (Brazil) were chosen for collecting macrophytes due to distinct geographic, climatic, and biome conditions. The first point was in Dourados (Site 1) at the Arnulpho Fioravante Park lagoon (S 22°14′03.10" and W 54°47′47.52"), located in the urban perimeter of the city and characterized by high eutrophication of the environment. The other site was in Corumbá (Site 2), on the Paraguay River, specifically at Porto Geral (S18°59′48'' and W 57°39'11''). Samples were collected on the banks of the sites.

The exsiccate collected from the Dourados sample was deposited under code DDMS 8511. The exsiccate collected from Corumbá was registered under code COR 18341. The authors confirm that the use of plants in the present study complies with international, national, and/or institutional guidelines. Both collections were registered under Code A51BA3A in Sistema Nacional de Gestão do Patrimônio Genético e do Conhecimento Tradicional Associado (SisGen).

The macrophytes were washed in running water and dried at room temperature (25 ± 1 °C) until a constant mass was obtained, which occurred over 7 days. Subsequently, the macrophytes were ground in a blender and sieved in a 0.5 mm mesh.

### Direct transesterification

Ethyl (EB) and methyl biodiesel (MB) were produced respectively from *E. crassipes* collected in the cities of Dourados (EBD and MBD) and Corumbá (EBC and MBC) utilizing the adapted methodology described in the literature^37^. On a 5.000 g sample of the dried plant, 17.00 mL of methanol, 20.00 mL of hexane, and 3.00 mL of concentrated sulfuric acid were added. The process was realized in triplicate. The resulting solutions were sealed and placed in the ultrasound at a frequency of 42 Hz (400 W) for 5 min to aid in the mechanical rupture of the cell membranes using vibrations. The process was also performed by substituting methanol for ethanol.

Next, the samples were placed under magnetic stirring for 60 min in a water bath at 90 °C. The reaction product was filtered on cotton and collected directly into a separation funnel. The retained biodiesel extraction of cotton biomass was done with 35.00 mL of hexane. The filtrate was placed in the separation funnel with 50.00 mL of distilled water for 30 min for phase separation. The supernatant was stored in test tubes and kept in the oven at 60 °C until constant mass and evaporation of any remaining solvent. The procedure was repeated, obtaining 65.00 g of biomass for methyl biodiesel and 50.00 g for ethyl biodiesel.

### Chromatographic analysis

The resulting biodiesel samples were analyzed for the amount and composition of fatty acid esters by gas chromatography in CG-FID (Thermo Scientific, USA) equipment. Resolutions ANP No. 45/2014 (https://www.gov.br/anp/pt-br/canais_atendimento/imprensa/noticias-comunicados/anp-publica-resolucao-sobre-especificacao-do-biodiesel), ASTM D6751 (https://www.astm.org/d0975-22a.html), and EN 14214 (European Standard EN 14214. Automotive fuels. Fatty acid methyl esters (FAME) for diesel engines. Requirements and test methods, 2008) to evaluate biodiesel with the specified quality control parameters.

The samples were injected using an injector (split 1:20) using a flame ionization detector. A SUPELCOWAX 10 capillary column (30 m length, 0.32 mm internal diameter, and 0.25 µm thickness) was used. The temperature ramp, consisting of 1 min at 130 °C, rises to 215 °C with a velocity of 7 °C min^−1^ and holds at 215 °C for 12 min, followed by a further rise to 250 °C with a rate of 20 °C min^−1^. The (hydrogen) entrainment velocity was kept constant at 1 mL min^−1^. The fatty acid content was obtained using the normalization method, and the result was expressed as percent relative. The unsaturation degree (UD) was calculated (Eq. 1) using the amounts of monounsaturated fatty acids (MUFA) and quantities of polyunsaturated fatty acids (PUFA) present in biodiesel^9^.

1 $$UD={W}_{MUFA}+\left({2W}_{PUFA}\right)$$

The average carbon number (ANC) was determined by Eq. (2), where C_n_ is the carbons of each fatty acid ester and w_i_ = weight percentage of each fatty acid ester.

2 $$ANC=\sum \left({nC}_{n}\times {w}_{i}\right)$$

The average number of double bonds (ANDBi) by Eq. (3), where LD is the number of double bonds^38^.

3 $$ANDBi=\sum \left(LD\times {w}_{i}\right)$$

### Biodiesel prediction analysis

The properties of biodiesel were simulated using BiodieselAnalyzer© Version 2.2 software^39^, which estimates the properties through calculations employing the fatty acid data.

### Statistical analysis

R version 4.0.3 software [R: The R Project for Statistical Computing (r-project.org)] was used in the statistical analysis. Regarding determining the normality of the repetitions, the Shapiro–Wilk and Bartlett tests were used to verify if the data were parametric. Subsequently, a one-way ANOVA analysis was performed to check if there was a significant difference between the treatments of the means of the yield, fatty acid ester content, and physicochemical properties separately. Finally, the Tukey test was used, with 5% significance, for the samples with a significance difference. Different letters were used to indicate significant differences between samples.

The Euclidean distance was used to assess the similarity between the samples, and a correlation dendrogram was plotted using the vegan package [CRAN—Package vegan (r-project.org)]. The Euclidean distance was employed in the other multivariate statistical analyses. To verify the influence of the collection site and alcohol used in direct transesterification, a Permutation Multivariate Analysis of Variance (PERMANOVA) was applied using the similarity of chemical composition by the vegan package. Finally, principal component analysis (PCA) was performed by the *FactoMineR*^40^* and factoextra* packages [CRAN—Package factoextra (r-project.org)].

## Data Availability

The datasets generated during and/or analyzed during the current study are available from the corresponding author on reasonable request (T. L. A. C.).
